# Apneic preoxygenation without nasal prongs: the “Hungarian Air Ambulance method”

**DOI:** 10.1186/s13049-016-0200-0

**Published:** 2016-01-21

**Authors:** Attila Eross, Laszlo Hetzman, Andras Petroczy, Laszlo Gorove

**Affiliations:** Hungarian Air Ambulance Nonprofit Ltd., Legimentok utca 8, Budaors, H-2040 Hungary; Department of Anaesthesiology and Intensive Care, Medical Centre, Hungarian Defence Forces, Robert Karoly korut 44, Budapest, H-1134 Hungary; Department of Anaesthesiology and Intensive Therapy, Semmelweis University, Kutvolgyi ut 4, Budapest, H-1125 Hungary

**Keywords:** Preoxygenation, Apneic preoxygenation, Apneic oxygenation, Prehospital, Helicopter emergency medical service, Intubation, Rapid sequence intubation

## Abstract

The Hungarian Air Ambulance has recently adopted oxygen supplementation during laryngoscopy, also known as apneic preoxygenation, to prevent desaturation during rapid sequence intubation. Despite its simplicity the nasal cannula method has some limitations relevant to our practice. First, the cannula can dislodge if the head is manipulated during preparation or intubation, especially if nasopharyngeal airways are chosen to maximise preoxygenation. Second, the method is incompatible with continuous nasal suctioning required in severe maxillofacial trauma. Third, if only one oxygen source and one competent assistant is available, a situation common during prehospital missions, the extra tube swap needed for continuous oxygen supplementation makes the procedure more complex and prone to error. We report a new method that provides comparable oxygen supplementation to the nasal cannula method, but at the same time eliminates the problems mentioned above and is easier and quicker to perform. It requires the intubator to cut and insert the tubing of the non-rebreather mask into the nasopharyngeal airway, thus providing direct pharyngeal insufflation. The method is applicable to every patient who has at least one nasopharyngeal airway inserted at the time of laryngoscopy and it only requires a pair of scissors.

**Electronic supplementary material:**

The online version of this article (doi:10.1186/s13049-016-0200-0) contains supplementary material, which is available to authorized users.

## Background

Nasopharyngeal [1–3] and nasal [4–6] oxygenation during apnea has been shown to prolong the onset of desaturation in normal and obese patients anaesthetized and paralyzed for elective surgery. Oxygen supplementation through a nasal cannula during laryngoscopy, also known as apneic preoxygenation, has recently been suggested by Weingart and Levitan [7] to prevent desaturation during rapid sequence intubation (RSI). The method was associated with decreased desaturation rates during RSI by Greater Sydney Area Helicopter Emergency Medicine Service (HEMS) [8].

The Hungarian Air Ambulance is the only HEMS in Hungary (population 9.9 million, area: 93.000 km^2^). It is a national, government funded service with seven bases, and it provides primary care for critically injured and ill adults and children on scene (case mix: 44 % trauma, 66 % medical, mean National Advisory Committee for Aeronautics (NACA) score in 2015 is 4.41). Units are staffed by a pilot, a paramedic and a physician (36 % anaesthetist, 51 % emergency physician, 13 % other speciality - 66 % consultant and 33 % registrar level). The Service performs approximately 200 RSIs a year using a standardized RSI system [9]. Apneic preoxygenation was incorporated into the RSI standard operating procedure in 2014.

## Main text

On introduction we identified several pitfalls with the nasal cannula method both during live missions and moulage scenarios. 1) First, it can be challenging to apply and keep the nasal prongs in place if nasopharyngeal airways (NPAs) are chosen to maximise preoxygenation, such as in patients with reduced level of consciousness. 2) Second, once applied any manipulation to the face and neck, such as chin lift or manual in-line stabilization, can easily dislodge the prongs from the nostrils or from the NPAs. 3) Third, the presence of blood, sometimes found in head or maxillofacial trauma, can easily obstruct the passage of oxygen. In severe cases continuous nasal suctioning is needed even during the laryngoscopy, however this is impossible without removing the nasal cannula. 4) Fourth, if only one oxygen source and one competent assistant is available, a situation common prehospitally, the assistant needs to ensure the tubing is swapped not just after the intubation (ie. to start supplementing the bag-valve), but after the induction as well (ie. to start apneic preoxygenation). As the tubing and cylinder are usually out of sight during the intubation, it is easy to forget about the second swap, thus making the patient prone to desaturation.

**Fig. 1:**
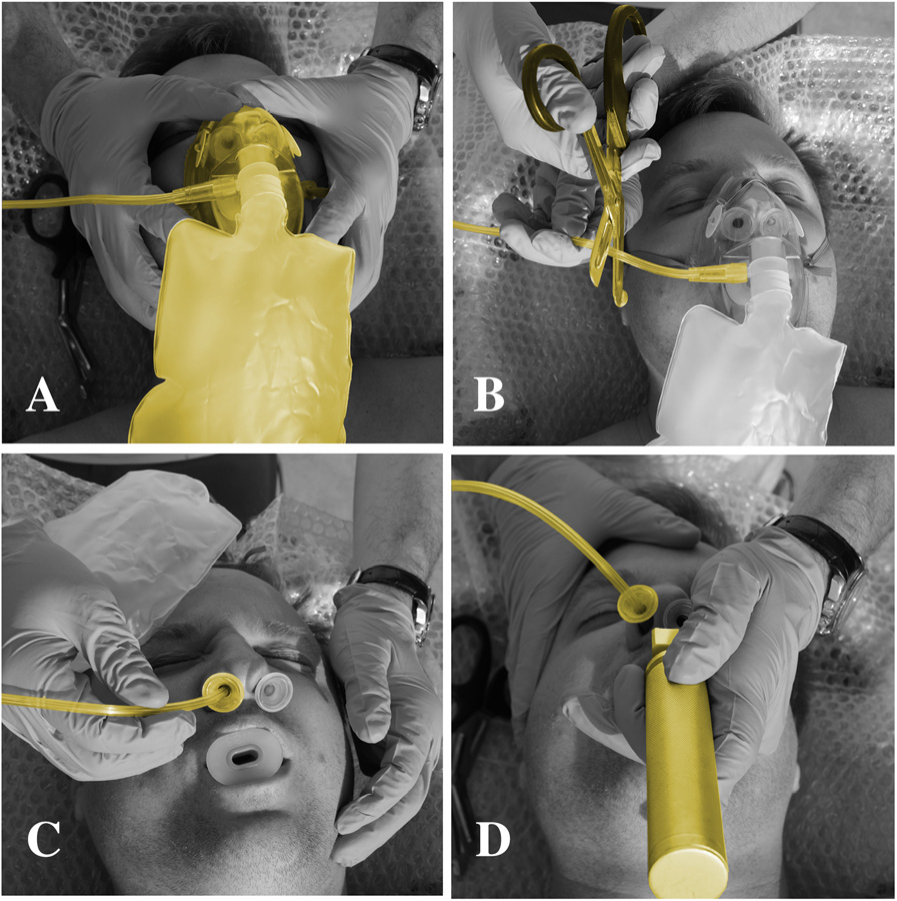
Apneic oxygenation without nasal prongs - the “Hungarian Air Ambulance method”. **a** The intubator preoxygenates the patient with a non-rebreathing mask (15 lpm). Upper airway patency is maximized by two naso- and one oropharyngeal airway. **b** The intubator cuts the tubing of the mask after the onset of apnea. **c** The intubator removes the mask and inserts the free end of the tubing approximately 3–5 cm deep into the nasopharyngeal airway. **d** The laryngoscopy is performed with the “tube in the tube”

We have developed a new method that eliminates the problems mentioned above and at the same time is easier and quicker to perform. It is applicable to every patient who has at least one appropriately sized, flinged nasopharyngeal airway inserted at the time of laryngoscopy. Preoxygenation is carried out in the standard way with a non-rebreathing mask (NRBM) set at 15 lpm (Fig. 1a). After the onset of apnea the intubator cuts the tubing of the NRBM (Fig. 1b), removes the mask and inserts the free end of the tubing approximately 3–5 cm deep into the nasopharyngeal airway (Fig. 1c) with the flow kept at 15 lpm. The laryngoscopy is performed with the “tube in the tube” (Fig. 1d). An additional movie file shows this in more detail [see Additional file 1].

Once the correct endotracheal position is confirmed, the assistant swaps the oxygen source, removes the tube from the NPA and the procedure is continued as usual. If the team has an extra cylinder the swap can be eliminated, and in case of a failed laryngoscopy with desaturation, the reoxygenation can be augmented by double oxygenation (ie. reservoir-bag-valve-mask plus nasal supplementation). In our experience the wall of the tube is rigid enough not to kink beneath the facemask. Should the patient have pharyngeal bleeding, continuous suctioning can be applied through the opposite NPA. This will most probably reduce the oxygen concentration within the pharynx, although it seems logical that it is still better than suctioning without oxygen supplementation.

One concern with nasopharyngeal oxygen insufflation is the risk of iatrogenic gastric rupture. A recent case report and literature search identified 19 cases since 1961 where oxygen was applied through nasopharyngeal or nasal catheter [10] in different clinical scenarios (respiratory insufficiency, procedural sedation or after operation). Patients were all breathing spontaneously. Flow rates, if reported, were usually 3 to 4 liters per minute. Time of oxygen therapy before the onset of symptoms was usually between several minutes to several hours. Possible mechanisms for stomach rupture included direct oesophageal insufflation (i.e. catheter placed or migrated to or below the level of the cricopharyngeus) or oxygen stream induced deglutition reflex resulting in persistent aerophagia. Air swallowing might have also been coupled by the suctioning effect of negative intrathoracic pressure from spontaneous breathing, exacerbated by the decreased tone of the oesophageal sphincter (direct drug effect) and/or the partial airway obstruction subsequent to the reduced level of consciousness. Given the fact that our method is 1) applied only for a short period of time (usually less than a minute), 2) the patient has no spontaneous breathing while applied and 3) there is no risk of catheter misplacement (i.e. the tubing needs to be introduced only a couple of centimetres in order to sit firm in the NPA), we believe the risk of iatrogenic gastric rupture is extremely small even with high flow and is undoubtedly outweighed by the benefit of preventing desaturation.

Since the introduction of the method, our Service has carried out 150 RSIs with apneic preoxygenation (91 trauma vs 59 medical, 112 men vs 38 women, mean age 51.9 [7–89]). The procedure was easy to perform in all cases and no complications were noted with its use. Desaturation (defined as peripheral oxygen saturation (SpO_2_) decreasing to or below 90 % during intubation) was observed in nine cases (6.0 %), and in three cases the providers noticed an increase of the SpO_2_ during the procedure. According to our previous study, 35 out of 433 patients (8.1 %) desaturated during a 29 months period before the introduction of apneic preoxygenation [9].

There are two limitations we have identified so far. 1) First, the method requires a NPA. We do not recommend passing the oxygen tube directly in the nasal passage, as it can provoke bleeding. In contrast to the soft and pliable NPA, oxygen tubing can penetrate the cranium through a basal skull fracture. 2) Second, the oxygen tube can not be inserted into pediatric NPAs due to its diameter, although this is not relevant in infants and small children where bag-mask ventilation is mandatory during the onset of muscle relaxation (‘modified RSI’).

Apart from these limitations we believe this simple “Hungarian Air Ambulance method” could be useful for those clinicians, that intubate patients requiring NPAs due to reduced level of consciousness and/or maxillofacial bleeding or any patient in whom NPAs can be inserted before or right after the induction of anesthesia.

## Conclusions

Despite its simplicity, apneic preoxygenation through a nasal cannula has some limitations (possibility of dislodgement, incompatibility with continuous nasal suctioning, requirement of an extra oxygen source). We report a new method that is applicable to every patient who has at least one nasopharyngeal airway inserted at the time of laryngoscopy. It requires the intubator to cut and insert the tubing of the non-rebreather mask into the nasopharyngeal airway, thus providing direct pharyngeal insufflation. The technique provides comparable oxygen supplementation, but at the same time eliminates the problems mentioned above and is easier and quicker to perform. It can be of interest to all clinicians dealing with acute airway management, and particularly colleagues working in low resource environment.

## Additional file

Additional file 1:
**Video demonstrating apneic preoxygenation without nasal prongs (Hungarian Air Ambulance method).** (MP4 11043 kb)

## Abbreviations

HEMS: helicopter emergency medicine service
NACA: National Advisory Committee for Aeronautics
NPA: nasopharyngeal airway
NRBM: non-rebreathing mask
RSI: rapid sequence intubation
SpO_2_: peripheral oxygen saturation
